# Knowledge, Attitudes and Perceptions towards Vitamin D in a UK Adult Population: A Cross-Sectional Study

**DOI:** 10.3390/ijerph15112387

**Published:** 2018-10-27

**Authors:** Clodagh O’Connor, Dominique Glatt, Lois White, Raquel Revuelta Iniesta

**Affiliations:** Dietetics, Nutrition and Biological Health Sciences, Queen Margaret University, Edinburgh EH21 6UU, UK; 16006436@qmu.ac.uk (C.O.); dglatt@qmu.ac.uk (D.G.); lwhite@qmu.ac.uk (L.W.)

**Keywords:** vitamin D, knowledge, attitudes, perceptions, fortification, supplementation

## Abstract

The prevalence of vitamin D deficiency in the United Kingdom is high, despite updated Scientific Advisory Committee on Nutrition (SACN) guidelines. Therefore, our aims were to identify population knowledge, attitudes and perceptions of vitamin D supplementation and factors contributing to supplement use in a UK adult population. A cross-sectional study was performed between April–June 2018 using a newly designed piloted questionnaire. Scores for knowledge were calculated as a percentage (Boland et al. 2015). Logistic regression analysis was used to predict supplement use. 209 participants (82% female), mean (±SD) age 34.9 (±12.3) completed the questionnaire. The mean (±SD) vitamin D knowledge score was 56.6% (±19.9%); only 48% were concerned about their vitamin D concentration and 57% did not take vitamin D. Most participants (86%) wished to learn more about vitamin D. Knowledge score (OR 2.5; *p* = 0.01; 95% CI 1.2–5.3), concern (OR 2.1; *p* = 0.03; 95% CI 1.0–4.2) and location (OR 0.3; *p* = 0.006; 95% CI 0.1–0.7) predicted supplemented use. Individuals living in England had 2.9 (95% CI 1.4–6.3) lower odds of taking vitamin D than those living in Scotland. As a result of these findings, this study suggests that vitamin D supplementation and fortification, alongside education strategies, may be an effective method for improving UK vitamin D health; however, more research is warranted.

## 1. Introduction

Plasma 25-hydroxivitamin D (25-OH-D) deficiency (<25 nmol/L) is a recognised health problem in the UK [1,2]. 25-OH-D is primarily obtained from Ultraviolet B (UVB) sunlight through dermal synthesis, but it can also be obtained from the diet. However, few foods naturally contain vitamin D, and in the UK fortification is rare [1]. The UK National Diet and Nutrition Survey (NDNS) from 2008–2012 identified poor vitamin D intake from food and supplement sources as well as reduced exposure to sun light [3]. Consequently, the Scientific Advisory Committee on Nutrition (SACN) updated guidelines to recommend vitamin D supplementation of 10 μg/day for the entire population (>5 years), particularly in winter (October–April) [4]. The mean vitamin D intake from dietary sources was 2.8 μg/day (112 International Units (IU)) in adults (19–65 years) and 3.3 μg/day (132 IU) in older adults (>65 years). Furthermore, 23% of adults and 21% of older adults in the UK were 25-OH-D deficient [3]. Additionally, previous NDNS reports showed supplement use resulted in a minimal increase in mean vitamin D intake from 2.8 μg/day (112 IU) to 3.6 μg/day (180) in adults (19–64 years) and from 3.3 μg/day (132 IU) to 5.3 μg/day (212 IU) in older adults (>65 years). This was attributed to compliance issues and the fact that few individuals take supplementation [3]. There are ongoing disagreements surrounding the definitions of vitamin D status and intake recommendations [4,5,6]; however, this article uses the status definitions presented by Holick et al. [5], as these are currently used by the UK government [4] and allow for comparisons with UK data.

It is well-established that vitamin D is essential for bone health, including regulating calcium metabolism and promoting intestinal calcium absorption [1,2]. Serum 25-OH-D concentration of less than 30 nmol/L has been associated with increased risk of rickets and reduced bone mass density in children and adolescents, increased risk of osteomalacia in young and middle aged adults and increased risk of osteoporosis and fractures in older adults [4,5]. Emerging evidence suggest vitamin D is required for non-musculoskeletal functions in the body [5,6,7]. A potential role in lowering blood pressure and reduce the risk of developing Diabetes Mellitus has been reported [5]. Work is also ongoing exploring its anti-inflammatory effects [4,7,8].

Despite 25-OH-D deficiency [6] and limited vitamin D intakes [3], there is a paucity of evidence concerning vitamin D related knowledge, beliefs and attitudes of the worldwide population [9,10,11,12,13,14,15,16,17,18,19,20] and that of the UK [10]. A small qualitative study performed in East London reported limited knowledge about the health benefits of vitamin D and confusion about both vitamin D food sources and the risks and benefits of sun exposure [10]. Likewise, populations from China [13], Australia [15] and Saudi Arabia [16], as well as France [17], had poor knowledge, despite having good vitamin D awareness. Exposure to sunlight was limited and attitudes towards vitamin D supplementation and fortification were mixed [10,11,12,13,14,15,16,17,18,19,20]. Of note, supplementation, fortification and factors contributing to vitamin D supplement use have rarely been investigated. Only unpalatability of vitamin D and calcium combined tablets [10] and poor knowledge [14] have been reported as barriers to supplement use in the UK and Canada respectively. Therefore, our aims were to investigate the knowledge, attitudes and perceptions of vitamin D supplementation and fortification and to identify potential factors contributing to vitamin D supplement use in a UK population. The expectation is that the results of this study will help develop effective clinical and public health strategies to improve vitamin D status.

## 2. Materials and Methods

### 2.1. Study Design, Population and Timeline

An observational, cross-sectional study was carried out from April to June 2018. Eligibility criteria included both male and female adults (>18 years) living in the UK to correspond with the NDNS adult population [3].

### 2.2. Questionnaire

A newly developed and piloted online questionnaire was employed to assess knowledge, attitudes and perceptions to vitamin D, sun exposure, fortification and supplementation of the UK population. This was developed based on previous studies investigating vitamin D knowledge [12,13,14,15], questions in current ongoing research [4], literature on vitamin D [1,2,3,4,5,6,7,8,9,10,11,12,13,14,15,16,17,18,19,20] and through consultation with academic Dietitians and Nutritionists. Questions were modified to align with current UK vitamin D recommendations [4] and be of relevance to the UK population. The questionnaire was created in an online format using the Bristol Online Survey tool [21] and consisted of 41 questions, each containing five sections; demographics, vitamin D knowledge, attitudes towards sun exposure, attitudes towards vitamin D, perceptions to food fortification and supplementation. At present, a validated questionnaire does not exist. Thus, this was piloted internally in a sample of Queen Margaret University (QMU) staff and students for content validity. Appropriate changes were made based on feedback. The completion of the questionnaire took no more than fifteen minutes (Supplementary Material 1) and was distributed to the UK population through QMU moderator emails and social media platforms (Facebook, Twitter, Instagram and LinkedIn), which included a direct link to the questionnaire and guidelines to consent.

Demographic data (age, gender, geographical location, education, ethnicity, skin type, height and weight and whether women are breastfeeding, pregnant or menopausal) was collected and continuous variables categorised. Age was categorised into < 65 and ≥ 65 as per NDNS report [3], body mass index (BMI) (kg/m^2^) was calculated from self-reported heights and weights and presented according to National Institute for Health and Clinical Excellence (NICE) (2014) criteria: underweight (<18.5), normal weight (18.5 to 24.9), overweight (25 to 29.9), obese (≥30). Ethnicity was further categorised into Caucasian and non-Caucasian and education into those with a university degree and those without to improve reliability of estimates in the analysis (Laerd Statistics 2015). In the education section, we asked to specify whether graduates had a nutrition related degree to account for bias and allow for comparisons with non-nutrition graduates.

For vitamin D knowledge, scores were calculated as per Boland et al. (2015) [14]. For those sections with only one correct answer, 1 point was granted per question. And for those sections with more than one correct answer, one point was granted per correct answer and one point was subtracted per incorrect answer. A total score was calculated, and the results were presented as a percentage [15]. Scores were then stratified into < 50% and > 50% to perform univariate association analysis between categorical variables and knowledge scores.

Sun duration variables were used to calculate the hours of exposure per week, by multiplying individuals sun exposure of 24 h per day by days per week, which was then dichotomised into ≥14 h/week and <14 h/week [15].

This study was granted ethical approval from QMU ethics committee in March 2018.

### 2.3. Statistical Analysis.

The Statistical Package for Social Science (IBM SPSS software; version 23, IBM, Armonk, NY, USA) was used to analyse all data. Figures were created in Microsoft Excel (Microsoft, Redmond, WA, USA).

Descriptive statistics were used to present demographic data and to evaluate knowledge, attitudes and perceptions of vitamin D supplementation and fortification. Univariate associations between categorical variables (demographics, nutrition qualification, knowledge score, university degree, location, concern about 25-OH-D levels, 25-OH-D tested and willingness to purchase/consume fortified foods) and supplement use were established by χ^2^-test. To establish factors contributing to supplement use the Binomial Logistic Regression Analysis was used. Factors with a *p*-value of *p* < 0.1 were included in the regression model. Results were expressed from the logistic regression using odds ratios, 95% confidence intervals and a *p* < 0.05 was considered statistically significant.

We followed the Strengthening the Reporting of Observational Studies in Epidemiology (STROBE) guidelines for the presentation and writing of our manuscript [22].

## 3. Results

In total, a convenient sample of 209 individuals participated in the study. The majority of participants were female (82%), under 65 years (96%), normal weight (52%), living in Scotland (60%), Caucasian (90%) and had a higher education qualification (76%). Participants’ demographics are summarised in Table 1.

### 3.1. Vitamin D Knowledge

All participants (99.5%), apart from one (0.5%), had heard of vitamin D. The most common source of vitamin D information was the media (25.3%), followed by friends/family (19.6%), health professionals (18.4%), university (13.1%), books (12.2%), leaflets/posters (6.5%) and “other” (4.9%), which included heath articles, personal research, work, school, and general knowledge.

**Figure 1:**
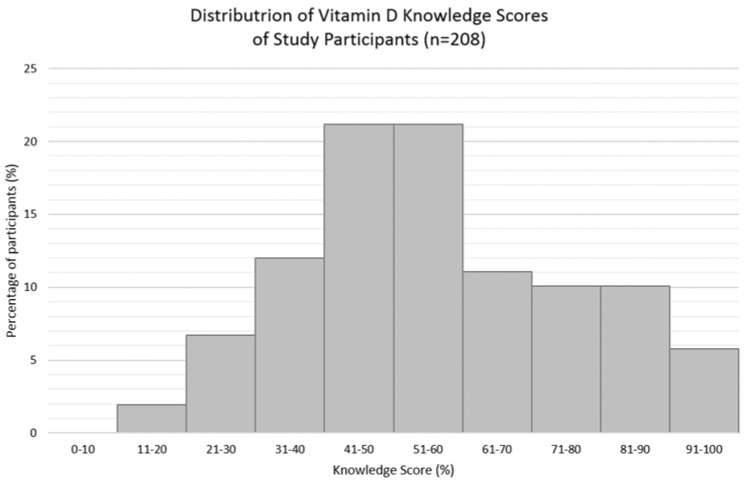
Overall knowledge scores of participants (*n* = 208, one participant did not respond).

**Figure 2:**
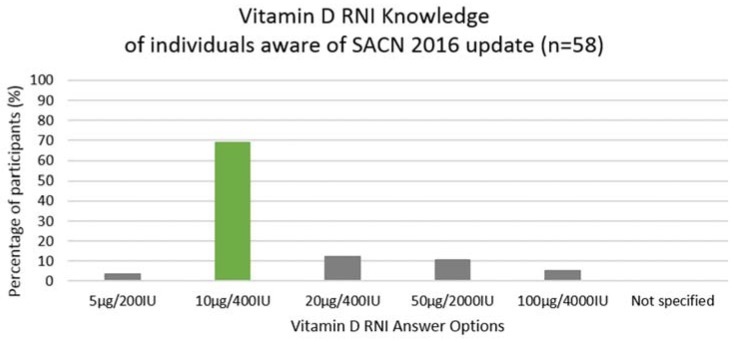
Vitamin D RNI knowledge of individuals aware of SACN 2016 guidelines. Participants’ (aware of the updated SACN 2016 vitamin D recommendations) knowledge on vitamin D RNI values (*n* = 58). Abbreviations: Reference nutrient intake (RNI); Scientific Advisory Committee on Nutrition (SACN).

The mean vitamin D knowledge score (±SD) was 56.0 ± 19.9%, and just over half (58%) of the participants scored > 50% (Figure 1). The mean vitamin D knowledge score (±SD) for nutrition students or graduates alone was 82.4 ± 11.2% (*n* = 40), and 50.6 ± 16.3% for those who identified as non-nutrition related studies (*n* = 165). There were statistically significant associations between knowledge scores (stratified into < and > than 50%) and knowledge sourced from university and family and friends. Those who scored ≤ 50% were less likely to have gained knowledge from university (χ^2^ = 38.910, *p* < 0.0005) and those who scored > 50% were less likely to have gained their knowledge from family and friends (χ^2^ = 5.947, *p* = 0.015). Participants’ knowledge of vitamin D are summarised in Table 2. The distribution of the participant’s vitamin D knowledge scores are presented in Figure 1 and Figure 2.

### 3.2. Perceptions and Practices towards Sun Exposure and Vitamin D

A total of 208 participant replies were recorded for the perceptions and practices towards sun exposure and vitamin D. Overall, 38% of participants (*n* = 80) were unsure whether ‘those with darker skin pigmentation were more at-risk of vitamin D insufficiency’, 38% (*n* = 79) agreed with that statement and 23% (*n* = 48) disagreed. A higher proportion (*n* = 104%, 50%) agreed that ‘skin pigmentations affected vitamin D status’, however, 40% (*n* = 83) were still unsure. Almost half of the participants (*n* = 99%, 48%) agreed that ‘if I regularly protected my skin from the sun, I may be in danger of not getting enough vitamin D’, while 32% (*n* = 67) disagreed and 18% (*n* = 38) were unsure (data not shown). Sun exposure practices are summarised in Table 3.

Sunscreen habits were queried of those participants who indicated that they “seek direct sunlight” when they go outside (*n* = 117) (Table 3); 31% answered they use sunscreen “sometimes” and 30% only used it for planned exposure or tanning. Eight percent said they used sunscreen “always”, 11% “usually”, 16% “rarely” and 5% “never” (data not shown). Of the 41 participants who indicated that they cover up when they go out (Table 3), 78% reported moderate coverage (exposure of forearms, below knee and face) and 22% reported maximal coverage (exposure of hands and face). There were no reports of minimal coverage (exposure of shoulders, above knee, and face) or total coverage (no skin exposure) (data not shown).

**Figure 3:**
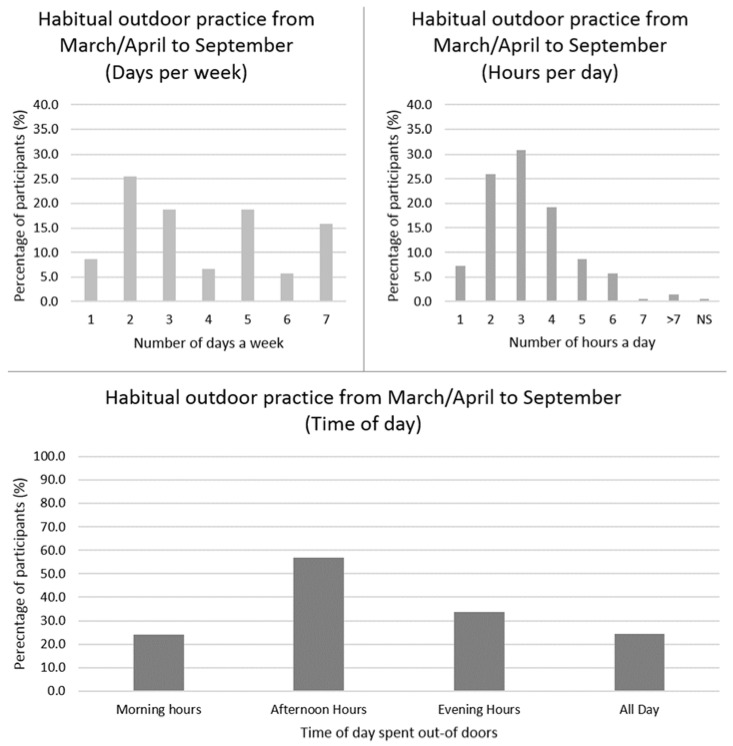
UK participants’ frequency of time spent outdoors in daylight from March/April to September (*n* = 208, one participant did not respond). Top left: Habitual number of days per week spent outdoors. Top right: Habitual number of hours per day spent outdoors. Bottom: Habitual time of day spent outdoors, not indicative of one single day; participants were able to select all relevant answers. Abbreviations: Not Specified (NS); Percentage (%).

On a weekly basis, 63% of the participants (*n* = 208) spent less than 14 h outdoors, with a mean (±SD) of 3.8 (±2.0) days per week and 3.2 (±1.4) h per day (data not shown). Habitual time spent outdoors is displayed in Figure 3.

### 3.3. Attitudes towards Vitamin D

Overall, 48% of participants (*n* = 208) indicated they were concerned their vitamin D levels may be too low, 39% were not concerned and 12% were unsure (one participant did not specify). The majority of participants (86%) were interested to know more about vitamin D. Only 13% of participants had had their vitamin D levels tested previously, while 5% were unsure. Of those who had had their vitamin D status tested (*n* = 26), 53% had done so due to healthcare professional advice, 27% due to concern regarding levels and 17% as a result of advice from friends or family. Only one participant had had their vitamin D status tested due to disease management.

### 3.4. Perceptions and Practices to Vitamin D Supplementation and Food Fortification

Only 43.5% (*n* = 91) of the study participants said they take a vitamin D supplement. The most commonly reported reasons for its use was insufficient sun exposure (57%), health benefits (51%), and insufficient amounts from food (46%). The perceptions of vitamin D supplementation are presented in Table 4.

**Figure 4:**
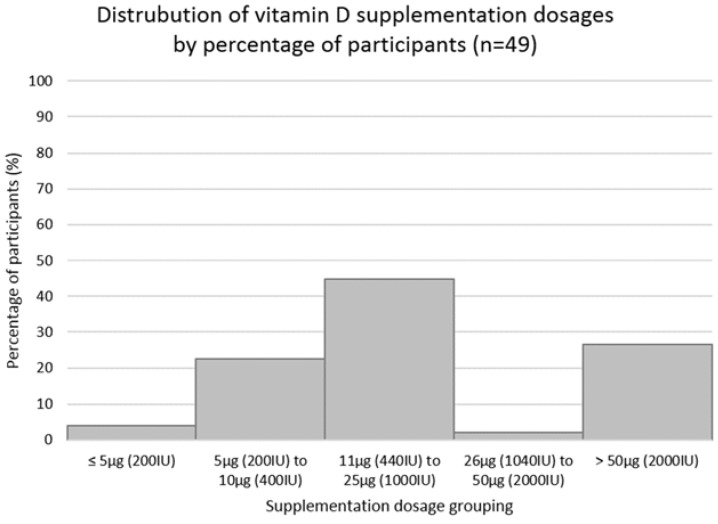
The distribution of vitamin D supplementation dosage by dosage groupings of the participants taking supplements and who know their dosage (*n* = 49). Abbreviations: Percentage (%); International Units (IU); micrograms (µg).

Of those taking vitamin D supplementation (91/209; 43.5%), 46% were unsure about the dose they were taking. Of those who knew (54%), 45% were taking vitamin D_3_ and 11% were taking vitamin D_2_. The most common mode of supplementation was using vitamin D capsules (52%), followed by multivitamin use (17%) and vitamin D drops (10%). Other modes included vitamin D oil, spray, cod liver oil, vitamin D and calcium combination tablets and other forms. The majority of participants took their supplements daily (77%), with the rest reporting a wide range of practices (including weekly (12%), less than weekly (5%), seasonally (1%), and other (5%)). Vitamin D supplementation dosages are displayed in Figure 4.

Perceptions on fortified foods varied amongst the participants (*n* = 208, one participant did not respond); 68% did not believe fortified foods were harmful; however, 24% were unsure and 8% believed they were. 65% of participants were willing to purchase or consume fortified foods, 24% were unsure and 11% were unwilling. Several reasons were given as to why the 16 participants felt that fortified foods were harmful: fear of vitamin overdose (*n* = 4%, 25%; prompted answer), lack of choice (*n* = 3%, 19%; prompted answer), a desire to avoid processed food products (*n* = 5%, 31%; free text answer), a belief that relying on fortified foods may eventually lead to less nutrition understanding (*n* = 2%, 12%), and a desire to achieve vitamin D status through natural sources (*n* = 2, 12%).

Supplement use logistical regression results are summarised in Table 5.

## 4. Discussion

This is the first study in the UK to quantitatively investigate the knowledge, attitudes and perceptions of vitamin D and the determinants of vitamin D supplement use. The results indicate that the UK population have a good level of knowledge with 99% correctly identifying the sun, 87% supplements and 84% food sources; however, specific knowledge of dietary food sources was poor. Attitudes towards vitamin D were mixed with only 50% of the population concern about their 25-OH-D levels. Notwithstanding, most individuals (86%) were willing to learn more about vitamin D. Furthermore, two thirds of the population would consume fortified foods, but vitamin D supplement use was poor (43.5%). Knowledge and concern about vitamin D were the main determinants of vitamin D supplement use and individuals living in Scotland were three times more likely to take a vitamin D supplement than those living in England.

### 4.1. Vitamin D Knowledge

In contrast to similar studies [13,14], we found higher mean knowledge score (56.6%) than Canada (27%) [14] and China 32.2% [13]. Other studies performed in England [10] and France [16] have both reported poor knowledge; yet the former was of qualitative nature and the latter did not report total scores. This difference may be due to our high representation of individuals with an undergraduate degree (40%), of which 20% was nutrition related, and higher representation of young females, who have previously been reported to have a better understanding of vitamin D [13,14]. Of note, this was not the case in England as older individuals were better informed [10]. Thus, we highlight the potential accuracy of information communicated in universities and inaccuracy to the general public.

The media was the most commonly reported source of information, which is in line with a study from England [10], but contrast with France [10], where the main source of information was physicians (41%). Importantly, this study found no associations between the media as a source of information and vitamin D knowledge, possibly indicating the mixed accuracy of information. Additionally, health professionals were not associated with knowledge scores highlighting the importance of developing strategies to improve education in this group and to ensure accurate information is communicated to the general public through both health care professionals and the media.

Overall knowledge on the three main vitamin D sources (sunlight, food and supplements) was good. In comparison to other studies, we found a higher proportion of participants correctly identifying sun (99% vs. 91%, 23%), supplements (87% vs. 74%, 0.9%) [13,14], and food (84% vs. 33%, 0.5%) [15,19] as sources. However, specific knowledge of dietary sources was poor; oily fish, considered the best dietary source of vitamin D, was only identified by half the population. A considerable proportion incorrectly reported dairy products and vegetables as sources, also reported by others [10]. Interestingly red meat, which according to the NDNS (2014) UK report is the largest contributor to vitamin D intake across age groups [3], was reported as a source by a minority. Vitamin D is naturally present in foods in limited amounts and while the majority of the population felt dietary sources were not sufficient to maintain 25-OH-D concentration, almost a third of participants believed they were sufficient or were uncertain. If individuals are unaware of the minimal dietary sources of vitamin D and also do not take supplements, vitamin D status will likely be poor during winter [9].

Our study showed that overall knowledge on bone health benefits of vitamin D was good (82%), which is in line with studies from India (88%) [18] and Vietnam (88.5%) [19]. However, a lesser proportion reported prevention of osteoporosis and rickets, potentially highlighting that although the majority have a good awareness of overall benefits to bone, a lesser amount associate this with the potential risks of deficiency.

Overall, participants have a moderately good knowledge of the effect of sunlight and various factors on vitamin D synthesis; a significant amount reporting season, skin pigmentation, sunscreen, time of day and cloud cover as barriers. A good awareness of 25-OH-D insufficiency during winter was observed. Considering seasonal effect, the majority correctly reported March/April to September as the best time for synthesis from sunlight, indicating awareness of insufficient vitamin D synthesis throughout winter. A lack of awareness was observed regarding SACN (2016) vitamin D recommendations of 10 μg supplementation [4], while only 69% of those who reported awareness choose the correct RNI [4]. Considering the difficulty in achieving vitamin D sufficiency, individuals are unlikely to achieve recommendations if they are unaware of them.

### 4.2. Attitudes towards Sun Exposure and Vitamin D

Our study showed that over half (56%) of the population have positive attitudes to sun exposure, as they would usually seek direct sunlight, wear sunscreen sometimes or plan for exposure/tanning and spend 12.2 ± 8.6 daylight mean hours per week outdoors, mostly in the afternoon. This suggests that the majority of the population receive sufficient vitamin D from sunlight during March/April to September. In contrast, other studies from China [13] and Vietnam [19] have reported negative attitudes to sun exposure; however, comparison with these other countries is difficult due to the cultural differences towards sunlight exposure and their preference for light skin colour [13]. Of note, we did not look at factors limiting sun exposure, such as long indoors working hours, which may reduce likelihood of adequate vitamin D synthesis. Instead, we assumed individuals willingness to seek direct sunlight.

Similar to China [13] and Australia [15], attitudes towards vitamin D were mixed with only half of participants concerned about poor 25-OH-D concentration. The populations’ awareness of 25-OH-D status was very poor, a minority (13%) had levels tested previously. This lack of current awareness and concern is likely contributing to poor concentration [20]; however, with individual willingness to learn more this should be used as a platform to further inform individuals.

### 4.3. Perceptions to Food Fortification and Vitamin D Supplementation

The majority of participants had positive attitudes to food fortification, willing to consume/purchase fortified food and believing that fortified foods were not harmful, contradicting a qualitative study from England [10]. These attitudes support the potential for investigating implementation of further fortification in the UK population [23].

Despite SACN’s [4] updated recommendations encouraging supplement use, we showed that vitamin D supplement use was poor (43.5%); nonetheless, higher than that reported by others < 35% [13,23,24]. Considering supplements are one of the most important sources due to limited sun exposure and dietary sources in the UK, we can suggest that this is potentially contributing to vitamin D deficiency. It is worth noting that supplement use is potentially underestimated as vitamin D capsules, instead of multivitamins, were the most common supplement type reported, which may be due to unawareness that multivitamins contain vitamin D [3]. Furthermore, as the study was carried out during the summer months, individuals who only consume supplements during winter may have been missed.

More than half of participants were aware of their supplement dosage, most commonly 10 μg (400 IU), showing, individuals taking supplements are taking appropriate dosages for sufficient 25-OH-D status [4]. Although the majority reported daily consumption, inconsistent consumption was reported due to forgetfulness while others only consumed supplements during winter, which may be contributing to modest increases in vitamin D intakes seen in individuals who consume vitamin D supplements [6].

### 4.4. Factors Contributing to Supplement Use

Individuals with better knowledge of vitamin D were 2.55 times more likely to take a vitamin D supplement, similarly observed by Boland et al. (2015) in Canada [14]. Interestingly, other studies [10,17] reporting poorer knowledge than ours also reported considerably lower supplement use in this group, which suggests that an improvement in vitamin D knowledge would lead to an increase in supplement use [17]. Individuals concerned with their 25-OH-D concentration were twice as likely to take a vitamin D supplement than those who were not concerned. This reflects the health belief model that individuals are more likely to act on a specific health practice if they believe the health threat to be serious [24].

Importantly, we found that individuals living in Scotland were three times more likely to take a vitamin D supplement than those living in England. Nonetheless, serum 25-OH-D concentrations are observed to be lower in Scotland compared to England and vitamin D public health guidelines from Scotland and England have the same recommendations [4]. An explanation for this could be our higher representation of Scottish graduate population and the lower synthesising UVB sunlight from a higher latitude [25].

### 4.5. Public Health Implications

The positive perceptions towards fortification, alongside poor knowledge on vitamin D dietary sources and poor supplement use, highlight the potential to investigate fortification strategies to improve intakes and status [23]. Recommendations of food vehicles have been made by other countries. For example, Canada, US and Finland to fortify milk, sour milk, yogurt with 0.5 µg/100 g (20 IU/100 g) and margarine with 10 µg/100 g (400 IU/100 g) vitamin D_3_ [26]. Whilst, in Germany, winter bread has been fortified with 11.3 µg/100 g (452 IU/100 g). Improvements in vitamin D intake were seen in 12–14 years old in Finland [26]; however, its efficacy has not been assessed yet; whilst in Canada those who consumed vitamin D fortified foods had 25-OH-D above 50 nmol/L [27]. Effective strategies should include looking at a range of products to reach all population sub-groups and prevent lack of choice previously reported as a barrier [28]. The level of fortification of foods also requires further investigation, a dose-dependent increase in 25-OH-D concentrations from vitamin D-fortified foods have been reported; however, frequency of dose requires consideration [12].

Our study proposes national public health messages across various platforms (i.e., media, health professional, universities, schools), especially through the media, to increase awareness among all sub-groups of the population. Considering a large proportion of the UK population are illiterate regarding health information [27,29,30,31,32], information currently available to the general population may be too complex and should be translated to simple, food-based terms. Consistent accurate information distribution on vitamin D is essential to avoid current confusion within the population and messages should be explained as clearly as possibly to avoid misinterpretation [29]. Although this study focused on adults, it is important that we investigate the information being conveyed to the whole population, particularly children and young people. Early childhood health promotion is thought to be essential for later adult health [30].

Widespread public health messages on the association between sun exposure and skin cancer risk are likely to be affecting attitudes to sun exposure and increase the likelihood of sun protection [33]. Messages on skin cancer prevention and benefits of sun exposure on 25-OH-D status should be clear and integrated [33].

### 4.6. Limitations & Future Research

We have identified several limitations related to the methodology of this study. In particular, the design of the questionnaire did not include any questions about working hours, so although we asked about exposure to sunlight and time spent outdoors, we have possibly overlooked any potential barriers to sun exposure. Secondly, a hard copy should have been distributed to reach more elderly (>65 years) individuals whose access to media may be limited. Therefore, future studies should allow the option to fill out a paper copy. Supplement use and types of supplements should be investigated in more detail. Future studies should also investigate seasonal differences in supplement use. Finally, sub-groups populations (nutrition students and graduates, pregnant or breastfeeding women, menopausal women, and individuals greater than 65 years of age) were very small and thus unrepresentative for further analysis. Future studies should target these populations.

Future research should validate this questionnaire to be able to perform another nationwide larger study taking into consideration the limitations discussed here. Importantly, serum 25-OH-D should be collected to be able to correlate knowledge, attitudes and perceptions with levels.

## 5. Conclusions

In conclusion, our study showed that both vitamin D fortification and supplementation were positively received amongst the study participants, which suggest that fortification and supplementation, alongside education strategies, may be an effective means to improve UK vitamin D health. Further research should investigate effective strategies to improve vitamin D knowledge, include a larger sample with representation of all subgroups of the UK population to confirm these findings and to validate the questionnaire used here.

## Figures and Tables

**Table 1:** Demographic characteristics of participants.

Demographic	Sub-Group	*n* (%)
**Age**	<65 years ≥65 years Not specified	201 (96) 6 (3) 2 (1)
	µ (±SD) (in years)	34.9 (±12.3) years
**WHO BMI Category**	Underweight (<18.5 kg/m^2^) Normal weight (18.5–24.9 kg/m^2^) Overweight (25.0–29.9 kg/m^2^) Obese (>30.0 kg/m^2^) Not specified	9 (4) 114 (54) 52 (25) kg/m^2^ 20 (10) 14 (7)
	µ (±SD) (in kg/m^2^)	24.5 (±4.3)
**Gender**	Female Male Not specified	171 (82) 35 (17) 3 (1)
**Female-Specific Factors**	Menopausal Breast-feeding Pregnant	19 (9) 4 (2) 2 (1)
**Location**	Scotland England Northern Ireland Wales Not specified	126 (60) 53 (25) 18 (9) 4 (2) 8 (4)
**Education Level Attained**	Undergraduate/Bachelor degree Master’s degree High school Postgraduate degree Other Doctoral degree Trade school Didn’t finish high school	84 (40) 53 (25) 32 (15) 14 (7) 14 (7) 8 (4) 3 (1) 1 (1)
**Nutrition-Related Qualification**	Yes No Not specified	41 (20) 165 (79) 3 (1)
**Ethnicity**	Caucasian (White) Asian Black/African American Latino Native Middle Eastern Not specified	187 (89.5) 8 (4) 2 (1) 2 (1) 2 (1) 1 (0.5) 7 (3)
**Skin Type**	Type 1—light/pale white Type 2—white/fair Type 3—white to moderate brown Type 4—moderate brown Type 5—brown/dark brown Type 6—very dark brown to black/black Not specified	27 (13) 84 (40) 79 (38) 12 (6) 1 (0.5) 1 (0.5) 5 (2)

All percentages out of a total of 209 participants. Missing values accounted for Abbreviations: Frequency (*n*); Percentage (%); World Health Organisation (WHO); Body mass index (BMI); Mean (µ); Standard deviation (SD).

**Table 2:** Participants’ knowledge of vitamin D (*n* = 208, one participant did not respond); 10 questions used in vitamin D knowledge score calculation.

	Answer	Correct (✓) Incorrect (✘)	*n* (%)
1. Overall vitamin D sources (multiple answers *):	Sun Supplement Food Exercise Air Water Don’t know	✓ ✓ ✓ ✘ ✘ ✘ ✘	206 (99) 181 (87) 175 (84) 1 (0.5) 0 (0) 0 (0) 1 (0.5)
**2. Best vitamin D source** **(one answer):**	Sunlight Food Supplement Don’t know Water Air Exercise	✓ ✘ ✘ ✘ ✘ ✘ ✘	181 (87) 13 (6) 9 (4) 5 (2) 0 (0) 0 (0) 0 (0)
**3. Vitamin D food sources** (multiple answers *):	Oily Fish Fortified foods Egg yolks Dairy products Vegetables Red meat Fruit Nuts Chicken Don’t know Not specified	✓ ✓ ✓ ✘ ✘ ✓ ✘ ✘ ✓ ✘ -	110 (53) 91 (44) 86 (41) 61 (29) 46 (22) 30 (14) 24 (11) 20 (10) 2 (1) 41 (20) 1 (0.5)
**4. Are dietary sources sufficient to maintain vitamin D levels** **(one answer):**	Yes No Unsure Not specified	✘ ✓ ✘ -	27 (13) 142 (68) 36 (17) 3 (1)
5. Factors affecting synthesis from sunlight (multiple answers *):	Season Skin Pigmentation Sunscreen use Time of day Cloud cover Latitude Pollution Smoking High-fat diet None of the above Don’t know	✓ ✓ ✓ ✓ ✓ ✓ ✓ ✘ ✘ ✘ ✘	137 (66) 121 (58) 114 (55) 101 (49) 100 (48) 72 (35) 42 (20) 26 (13) 18 (9) 3 (2) 32 (15)
6. Vitamin D health benefits (multiple answers *):	Bone health Prevention of osteoporosis Prevention of rickets Skin health Hair growth Vision None of the above Don’t know	✓ ✓ ✓ ✘ ✘ ✘ ✘ ✘	171 (82) 144 (69) 121 (58) 76 (37) 44 (21) 37 (18) 4 (2) 15 (7)
**7. Aware of updated UK vitamin D recommendations (one answer):**	No Yes Not Specified	✘ ✓ ✘	146 (70) 58 (28) 4 (2)
**8. Vitamin D RNI (one answer):**	5 µg/200 IU 10 µg/400 IU 20 µg/800 IU 50 µg/2000 IU 100 µg/4000 IU Not specified	✘ ✓ ✘ ✘ ✘ ✘	14 (7) 81 (39) 51 (25) 31 (15) 23 (11) 8 (4)
9. Individuals most at risk of vitamin D deficiency (multiple answers *):	Individuals not outdoors often Institutionalised individuals Cover up skin when out Individuals with dark skin Individuals who don’t eat fish None of the above Not Specified	✓ ✓ ✓ ✓ ✘ ✘ ✘	183 (88) 142 (68) 130 (63) 61 (29) 35 (17) 4 (2) 12 (6)
**10. Time of year able to receive sufficient synthesis (one answer):**	March/April to September All-year October to March Unsure	✓ ✘ ✘ ✘	163 (78) 30 (14) 1 (1) 14 (7)

* Expresses percentages represent number of participants (*n* = 208) who selected the indicated answer Abbreviations: Frequency (*n*); Percentage (%); Correct answer option (✓); Incorrect answer option (✘); Reference nutrient intake (RNI).

**Table 3:** UK participants’ sun exposure practices from March/April to September (*n* = 208, one participant did not respond).

	Answers	*n* (%)
**Usual practice in sunlight:**	Seek direct sun Shade Cover-up/wear clothing Don’t go outside	117 (56) 50 (24) 41 (20) 0 (0)
**Frequency of sunscreen use/sun protection:**	Sometimes Only for planned exposure/tanning Usually Rarely Always Never	69 (33) 51 (25) 32 (15) 31 (15) 19 (9) 6 (3)

Abbreviations: Frequency (*n*); Percentage (%).

**Table 4:** UK participants’ perceptions of vitamin D supplementation (*n* = 209).

Question	Answers and Sub-Categories	*n* (%)
Reasons for not taking supplement (*n* = 118; multiple answer selection possible ^∧^):	“I think I get enough”	43 (36)
	Unaware of benefits of taking them	30 (25)
	“I don’t know which one I should take”	26 (22)
	“I don’t think it’s important”	10 (9)
	Too expensive	5 (4)
	“I don’t’ know how I can get them”	3 (3)
	Other (additional participant reasons)	
	Forgetful/lazy/busy	12 (10)
	Unnatural/dislike taking tablets	3 (3)
	Unwell after use	2 (2)
	Lack of research on benefit	1 (1)
	Other	4 (3)
	Non-specified	3 (3)
Reasons for taking supplement (*n* = 91; multiple answer selection possible *):	Don’t get enough from sun exposure	52 (57)
	Good for my health	46 (51)
	Don’t get enough from food	42 (46)
	Healthcare professional advice	22 (24)
	Updated guidelines recommendations	18 (20)
	Other (additional participant reasons):	
	Friend/family advice	13 (14)
	Mood/Seasonal Affective Disorder	4 (4)
	Other	6 (7)

Abbreviations: Frequency (*n*); Percentage (%); United Kingdom (UK). ^∧^ Expresses percentage of participants not taking supplements (*n* = 118) who found reason for not taking supplement applicable. * Expresses percentage of participants taking supplements (*n* = 91) who found reason for taking supplement applicable.

**Table 5:** Factors contributing to vitamin D supplementation use. A logistic regression predicting likelihood of supplement use on relevant factors (*n* = 208, one participant did not respond).

	B	SE	Wald	df	*p*	Odds-Ratio	95% CI for Odds-Ratios	
							*Lower*	*Upper*
Nutrition-related qualifications *Those with qualification compared to those without*	0.223	0.445	0.252	1	0.616	1.250	0.523	2.990
Knowledge score *Higher scores (>*50%*) compared to lo*wer (≤50%)	0.934	0.372	6.300	1	**0.012** *	2.545	1.227	5.279
University degree *Individuals with higher education degree compared to those without*	0.458	0.413	1.228	1	0.268	1.581	0.703	3.555
Location (1) *Wales compared to Scotland*	−1.067	1.386	0.593	1	0.441	0.344	0.023	5.200
Location (2) *England compared to Scotland*	−1.076	0.394	7.465	1	**0.006** *	0.341	0.158	0.738
Location (3) *Northern Ireland compared to Scotland*	−0.886	0.612	2.094	1	0.148	0.412	0.124	1.369
Concern re levels (1) *Individuals who are “unsure if concerned” compared to those who “are not concerned”*	0.055	0.631	0.008	1	0.930	1.057	0.307	3.641
Concern re levels (2) *“Yes” compared to “no”*	0.750	0.359	4.365	1	**0.037** *	2.118	1.048	4.281
Levels tested (1) *“Unsure” compared to “no”*	−0.646	0.808	0.640	1	0.424	0.524	0.108	2.553
Levels tested (2) *“Yes” compared to “no”*	0.757	0.510	2.204	1	0.138	2.131	0.785	5.785
Willingness to purchase/consume fortified foods (1) *“Unsure” compared to “no”*	−0.654	0.626	1.091	1	0.296	0.520	0.153	1.773
Willingness to purchase/consume fortified foods (2) *“Yes” compared to “no”*	0.109	0.541	0.041	1	0.840	1.116	0.386	3.221
Constant	−1.198	0.621	3.719	1	0.054	0.302		

Abbreviations: B coefficient (B); Standard Error (SE); Wald chi-square test (Wald); degrees of freedom (df); *p*-value (*p*); Confidence Interval (CI). * significance taken at *p* < 0.05 (in BOLD).

## Supplementary Materials

The following are available online at http://www.mdpi.com/1660-4601/15/11/2387/s1, Figure S1: title, Table S1: title, Video S1: title.
