# Supplementary material for: Knowledge, Attitudes and Perceptions towards Vitamin D in a UK Adult Population: A Cross-Sectional Study
Source: Int J Environ Res Public Health. 2018 Oct 27;15(11):2387. doi: 10.3390/ijerph15112387 (PMC6267199; doi:10.3390/ijerph15112387)
Supplement: Supplementary file 1 [file ijerph-15-02387-s001.pdf]

Supplemental Material 1

Questionnaire

Vitamin D knowledge, perceptions and practices questionnaire

Note: For questions asking for "all correct answers", correct answers indicated in ***BLOLD****.*

Section 1: Demographics

1. What age are you?

_____ years

2. Are you:

☐ Male
☐ Female

3. Where do you currently live?

☐ Scotland
☐ Wales
☐ England
☐ Northern Ireland

4. Are you currently menopausal, breastfeeding or pregnant?

☐ Yes
☐ No

- If you selected yes, please specify:

☐ Menopausal
☐ Pregnant
☐ Breast-feeding

5. What is the highest level of education you have completed?

☐ Didn't finish high school
☐ High school
☐ Trade School
☐ Undergraduate/Bachelor's degree
☐ Postgraduate degree (PGDip)
☐ Master's degree
☐ Doctoral degree (PhD)
☐ Other

- If you selected other, please specify: ____________________________
- If you selected undergraduate/Bachelor's, please specify: ____________________________
- If you selected postgraduate degree (PGDip), please specify: ____________________________
- If you selected master's degree, please specify: ____________________________
- If you selected doctoral degree (PhD), please specify: ____________________________

6. Do you have any nutrition-related qualifications or are you studying to get a nutrition-related qualification (i.e. BSc/MSc dietetics or nutrition)?

☐ Yes
☐ No

- If you selected yes, please specify: ______________________

7. Which ethnicity best describes you?

☐ Caucasian (white)
☐ Black or African America
☐ Asian
☐ Middle eastern
☐ Latino
☐ Native American

8. Based on the Fitzpatrick Scale, which skin type best describes yours? (choose ONE of the following)

☐ Light or pale white- Always burns, never tans
☐ White, Fair- Usually burns, tans with difficulty
☐ Medium, between white to moderate brown- Moderately burns, moderately tans
☐ Moderate brown- Rarely burns, tans more than average
☐ Brown, dark brown- Rarely burns, tans very easily
☐ Very dark brown to black, black- Never burns, tans very easily, deeply pigmented

9. What height are you?

____

- Specify units:
  ☐ feet and inches ☐ m ☐ cm

10. What weight are you?

____

- Specify units:
  ☐ stone (st) and pounds (lbs) ☐ lbs ☐ kg

Section 2: Vitamin D Knowledge

11. Have you previously heard of vitamin D?

☐ Yes
☐ No

12. If yes, where did you receive this information? (Select ONE OR MORE of the following)

☐ Health professionals (doctor, nurse, dietitian, nutritionist)
☐ University
☐ Media (TV, newspaper, radio, internet, magazine)
☐ Book
☐ Leaflets/Posters
☐ Family/Friends
☐ Other

- If you selected other, please specify: ________________________

13. Which of the following are sources of vitamin D? (select ONE OR MORE of the following)

☐ I don’t know
☐ **Food**
☐ **Supplements**
☐ **Sunlight**
☐ Water
☐ Air
☐ Exercise

14. What is the best source of vitamin D? (please select ONE answer)

☐ I don’t know
☐ **Sunlight**
☐ Food
☐ Water
☐ Air
☐ Exercise
☐ Supplement

15. What are the best food sources of vitamin D? (select ONE OR MORE of the following)

☐ I don’t know
☐ **Oily fish**
☐ **Egg yolks**
☐ **Fortified foods**
☐ **Red meat**
☐ Dairy products
☐ Fruit
☐ Vegetables
☐ Chicken
☐ Nuts

16. Do you think dietary sources are sufficient to maintain vitamin D levels?

☐ Yes
☐ No
☐ Unsure

17. Vitamin D can be produced/synthesized in the skin, what factors affect this vitamin D production/synthesis? (select ONE OR MORE of the following)

☐ I don’t know
☐ **Skin pigmentation**
☐ **Cloud cover**
☐ **Pollution**
☐ **Time of day**

- ☐ **Latitude**
- ☐ **Season**
- ☐ Smoking
- ☐ **Sunscreen use**
- ☐ High-fat diet
- ☐ None of the above

18. According to research, what are the health benefits of vitamin D? (select ONE OR MORE of the following)

- ☐ I don't know
- ☐ **Bone health**
- ☐ **Prevention of rickets**
- ☐ Vision
- ☐ Hair growth
- ☐ Skin health
- ☐ **Prevention of osteoporosis**
- ☐ None of the above

19. Are you aware of the new updated vitamin D recommendations in the UK?

- ☐ Yes
- ☐ No

20. What is the recommended daily amount of vitamin D supplementation for adults? Where µg = microgram and IU = International units (please select ONE answer)

- ☐ 5µg/200IU
- ☐ **10µg/400IU**
- ☐ 20µg/800IU
- ☐ 50µg/2000IU
- ☐ 100µg/4000IU

21. Who are most at risk of vitamin D deficiency? (select ONE OR MORE of the following)

- ☐ I don't know
- ☐ **Individuals not often outdoors (i.e. not out during daylight hours)**
- ☐ **Institutionalized individuals (i.e. care home)**
- ☐ **Individuals who cover up majority of their skin when outdoors**
- ☐ **Individuals with dark skin**
- ☐ Individuals who don't eat fish
- ☐ None of the above

22. When do we get sufficient Vitamin D synthesis from sunlight exposure in the UK? (please select ONE answer)

- ☐ All Year
- ☐ **March or early April to September**
- ☐ October to March
- ☐ Unsure

Section 3: Attitudes towards Sun Exposure

23. Those with darker skin pigmentation are more at-risk of Vitamin D insufficiency

☐ Agree
☐ Unsure
☐ Disagree

24. Skin pigmentation affects vitamin D status

☐ Agree
☐ Unsure
☐ Disagree

25. If I regularly protect my skin from the sun, I may be in danger of not getting enough vitamin D

☐ Agree
☐ Unsure
☐ Disagree

26. When spending time outdoors in the sunlight during March to end of September, do you usually:

☐ Seek direct sun
☐ Shade
☐ Cover-up or wear clothing
☐ Don't go outside

- If you chose to cover up, how do you cover up (choose closest match to habitual attire)?
  - ☐ Minimal coverage (exposure of shoulders and above the knee)
  - ☐ Moderate coverage (exposure of forearms, below knee and face)
  - ☐ Maximal coverage (exposure of only hands and face)
  - ☐ Total coverage (no skin exposure)

27. When sunny from March to end of September, how often do you wear sunscreen/sun protection?

☐ Never
☐ Rarely
☐ Usually
☐ Always
☐ Sometimes
☐ Only for planned exposure/tanning

- If never why not?

  ____

28. Regarding typical daylight exposure from the March until end of September, how many days per week on average would you spend outdoors (Please select ONE answer that best describes average sun-exposure)

☐ 1 day ☐ 2 days ☐ 3 days ☐ 4 days ☐ 5 days ☐ 6 days ☐ 7 days

29. On these days of daylight exposure, how long on average would you spend outside each day?

☐ 1 hour ☐ 2 hours ☐ 3 hours ☐ 4 hours ☐ 5 hours ☐ 6 hours

☐ 7 hours ☐ more than 7 hours

30. On these days of daylight exposure, what time of the day would you most often be out? (select all that apply)

☐ All day
☐ Evening hours
☐ Afternoon hours
☐ Morning hours

Section 4: Attitudes towards vitamin D

31. Are you concerned that your vitamin D levels may be too low?

☐ Yes
☐ No
☐ Unsure

32. Have you ever had your vitamin D levels tested?

☐ Yes
☐ No
☐ Unsure

- If yes, why?

☐ Healthcare professional advised me to
☐ Concerned about levels
☐ Friend or family advised it
☐ Other

If you selected other, please specify:

____

33. Are you interested to know more about vitamin D?

☐ Yes
☐ No

Section 5: Perceptions to food fortification and supplementation

34. Do you think there is any harm in taking fortified foods?

☐ Yes
☐ No
☐ Unsure

- If yes, why?

☐ Fear of vitamin overdose
☐ Lack of choice
☐ Other

If you selected other, please specify:

____

35. Would you be willing to purchase/consume fortified foods?

☐ Yes
☐ No
☐ Unsure

36. Do you take a vitamin D supplement?

☐ Yes
☐ No
-

37. If yes, why?

☐ Healthcare professional advised me too
☐ Good for my health
☐ Updated guidelines recommended me
☐ I don't think I get enough from food
☐ I don't think I get enough sun exposure
☐ Friend and/or family advised me
☐ Other

If you selected other, please specify:
____

38. If yes, which type of vitamin D supplementation do you use?
(select ONE OR MORE of the following)

☐ Vitamin D capsule
☐ Vitamin D oil
☐ Vitamin D drops
☐ Combined vitamin D and calcium tablet
☐ Multivitamin
☐ Cod liver oil
☐ Other

If you selected other, please specify:
____

39. If yes, how often do you take your vitamin D supplement?

☐ I don't know
☐ Daily
☐ Weekly
☐ Less than weekly
☐ Other

If you selected other, please specify:
____

40. If yes, without referring to the supplement packaging, are you aware of the dosage of vitamin D you receive from each supplement consumption?

☐ Yes
☐ No

If yes, what is the exact dosage?
___________________________ ☐ International units (IU) ☐ microgram (μg)

41. If no, why not?

☐ Unaware of the benefits of taking them
☐ Too expensive

☐ I don't know which one I should take
☐ I don't know how I can get them
☐ I think I get enough
☐ I don’t think it's important
☐ Other

If you selected other, please specify:

____
